# Incidence, time trends and predictors of acute kidney injury in the region of Stockholm, Sweden

**DOI:** 10.1038/s41598-026-67110-y

**Published:** 2026-08-15

**Authors:** Jim Alkas, Carl-Gustaf Elinder, Peter Barany, Arvid Sjölander, Juan-Jesus Carrero

**Affiliations:** 1 Department of Medical Epidemiology and Biostatistics, Karolinska Institutet, Stockholm, Sweden; 2 Division of Nephrology, Department of Clinical Science, Intervention and Technology, Karolinska Institutet, Stockholm, Sweden; 3 Division of Nephrology, Department of Clinical Sciences, Karolinska Institutet, Danderyd Hospital, Stockholm, Sweden

**Keywords:** Acute kidney injury, Chronic kidney disease, Albuminuria, Hypertension, Diabetes mellitus, COVID-19, Diseases, Medical research, Nephrology, Risk factors

## Abstract

Acute kidney injury (AKI) is a common and potentially serious complication, yet population-level data on its burden and determinants remain limited. We quantified AKI incidence, temporal trends, and risk factors across a complete healthcare system. We conducted a population-based cohort study including all adult residents of Stockholm, Sweden (2017–2021) using the SCREAM project. AKI events requiring hospitalization or occurring during hospital stays were identified using a composite algorithm incorporating laboratory-based KDIGO creatinine criteria, ICD-10 diagnoses, and acute dialysis procedure codes. We calculated annual incidence rates, temporal trends using Poisson regression, and incidence rate ratios for selected demographic, clinical, and laboratory-defined risk factors. Among approximately 1.9 million adults annually, 0.6–0.7% experienced AKI each year (71–95 per 10,000 person-years). AKI occurred in 5.8–7.8% of hospitalizations, with only 30% reaching KDIGO stage 2–3. From 2017 to 2021, AKI incidence increased by 26% (95% CI 1.23–1.29). Populations at highest risk of AKI were largely consistent over the years, and included older adults, men, and individuals with uncontrolled diabetes, prior AKI, hypertension, heart failure, kidney transplantation, and multiple myeloma. Lower eGFR and higher albuminuria were associated with markedly higher AKI risk in a graded manner. COVID-19 infection was associated with a six-fold higher AKI risk in 2020, but marked attenuation in 2021 suggests it does not explain the overall upward trend. This region-wide analysis quantifies the contemporary burden of AKI and identifies high-risk populations that may benefit from targeted prevention and monitoring strategies.

**Supplementary Information:**

The online version contains supplementary material available at 10.1038/s41598-026-67110-y.

## Introduction

Acute kidney injury (AKI), defined as a sudden deterioration in kidney function over hours to days, is a common condition, occurring in an estimated 8.4–17.6% of all hospital admissions^1^. It is associated with poor in-hospital outcomes^2,3^, prolonged hospital stays^3^, increased health care costs^2,4,5^ and higher mortality^3^. Among patients who survive to discharge, the long-term risk of adverse outcomes remains substantial, including rehospitalization with heart failure^6^, progression to chronic kidney disease (CKD) or end-stage kidney disease (ESKD)^7,8^, and death^9,10^.

Despite its clinical importance, the burden of AKI within entire healthcare systems remains insufficiently characterized. Prior studies have primarily examined AKI incidence in specific contexts such as major surgery (cardiac and non-cardiac)^10,11^, sepsis^12,13^ or intensive care unit (ICU) settings^8,14^. Better knowledge of the population-level burden of AKI, and the identification of groups at highest risk^15,16^, are of value to guide resource allocation, early detection, and initiation of prevention strategies. However, few investigations to date have assessed the full population impact of AKI across the healthcare continuum.

Although assessment of kidney function is recommended routinely prior and after surgical procedures^17^, transient creatinine elevations are often overlooked and may not be recognized with a clinical diagnosis of AKI. The development of standardized consensus definitions based on acute creatinine changes, rather than reliance solely on diagnostic codes, has improved the ability to quantify the burden and consequences of AKI in epidemiological studies^18,19^. Using this lab-based approach, 3 population-based studies reported relatively stable AKI incidence over the past two decades across diverse study periods and geographical settings: 2011–2014 in the UK, Canada, and Denmark^20^; 2013–2017 in a U.S. Veterans health system^21^; and 2006–2014 in a U.S. tertiary care hospital (Mayo Clinic Hospital)^22^.

In this study, we set up to comprehensively evaluate the burden and temporal trends of AKI incidence in the region of Stockholm over a five-year period and identified major conditions associated with its onset. Our clinical objective is to inform on patient subgroups at risk of experiencing AKI and that may benefit from preventative and monitoring strategies. As unique strengths, we captured the complete healthcare of the region, including laboratory tests, diagnoses and procedures which allowed us to improve the capture and ascertainment of AKI events. We hypothesize that new triggers, such as the COVID-19 (SARS-CoV-2) pandemic may have impacted on AKI trends.

## Methods

### Data source

The Stockholm CREAtinine Measurements (SCREAM) project is healthcare-utilization cohort including all residents in the region of Stockholm^23^. The region of Stockholm is the most populated area in Sweden, capturing about 25% of the total Swedish population. SCREAM captures and links healthcare data since 2006. Linkage with a variety of government-run databases, using the unique personal identification number of each inhabitant in Sweden, allowed precise ascertainment of the arrival/departure from the census of the region (both migration and death), and extraction of all laboratory tests, issued clinical diagnoses, and dispensed medications. The study was approved by the National ethics board (application number 2017/793 − 31) and the Swedish National Board of Welfare, with a waiver of informed consent because data made available to us was de-identified. Data were stored and analyzed according to the European Union General Data Protection Regulation (GDPR) law and Karolinska Institutet regulations for documentation and archiving. All methods were carried out in accordance with the Declaration of Helsinki.

### Study design

We created five calendar year cohorts (one for each year during the 5 most recent years available to us, that is 2017–2021) including all adult residents (≥ 18 years) registered in the population census of the region of Stockholm on January 1st of each year, which was the cohort entry date. The entire region population of each year was then observed up to Dec 31st, censoring at death or emigration. Within this design, participants could be naturally included in different calendar year cohorts, and the sole exclusion criterion applied was undergoing maintenance dialysis.

### Study covariates

At cohort entry, we collected data for all Stockholm citizens regarding 39 potential conditions that in previous literature have been linked to AKI, including demographics (age, sex, and highest attained education level), kidney function by eGFR level, albuminuria and a variety of comorbid conditions and procedures (Listed in Supplementary Table S1). Key comorbidities included diabetes mellitus, hypertension, kidney transplant status, cancer, previous myocardial infarction, heart failure, liver disease, multiple myeloma, and rheumatic disease. We did not include pharmacologic exposures that could precipitate or prevent AKI; our objective was to identify populations inherently at risk rather than the consequences of specific transient treatments.

We extracted outpatient measurements of serum/plasma creatinine (SCr/PCr) performed in the year preceding index date and used creatinine to estimate eGFR using the 2009 CKD-EPI (Chronic Kidney Disease Epidemiology Collaboration) equation (24). Creatinine values < 25 umol/L were considered implausible and excluded. We also extracted outpatient measurements of albuminuria, which has been suggested to predict AKI occurrence in previous studies^15^. All methods of albuminuria testing were considered: dipstick albuminuria or proteinuria tests, 24-h and spot albumin concentrations, urinary protein-to-creatinine ratio and urinary protein or albumin-to-creatinine ratio (UACR). Urinary protein-to-creatinine ratio and dipstick tests were approximated to UACR values using previously validated equations (25) and then categorized (A1, < 30 mg/g; A2, 30–300 mg/g; A3, > 300 mg/g) according to the KDIGO classification^26^. Some participants did not have SCr/PCr or albuminuria tested within the loop-back periods of our study (one year prior). This data could not be considered missing at random given the indications for testing behind these laboratory measurements. Thus, we opted for adding a “missing” category if absent. Some patients had multiple measurements of creatinine in the year prior. Following the approach of recent publications^27,28^, we used the mean creatinine value, on the assumption that most likely represents the kidney function of the patient on stable conditions. For patients with multiple albuminuria tests in the year prior, we prioritized measurements of UACR, and if only dipstick is available, we chose the conservative scenario of the most severe A category. Besides comorbidities, we obtained data of acute COVID-19 infection using the first positive polymerase chain reaction (PCR) or antigen test recorded from the Swedish Public Health Agency.

### Study outcome

The study outcome was the occurrence of AKI resulting in hospitalization or occurring during a hospital stay. AKI events were ascertained through difference data sources, including clinical diagnoses (ICD-10 code N17), need of acute dialysis procedure codes (DR015, DR023) and transient creatinine elevations following the KDIGO 2012 definition^29^ (Table 1). For the evaluation of acute creatinine increases we extracted all performed creatinine determinations within the preceding 365 days and during the stay of each hospitalization. During hospital stay we considered serum/plasma creatinine tests as well as those from arterial and venous blood gas methods from emergency and intensive care units. The median value of all outpatient creatinine tests in the 1-365 days prior to hospital admission was used as the “baseline creatinine”, in accordance with previous studies^1,20^. The 2012 KDIGO definition of AKI (Table 1) was applied to identify and classify AKI events, based on either a ≥ 1.5-fold increase from baseline serum/plasma creatinine or, when a baseline measurement was missing, an absolute increase in serum creatinine of ≥ 26.5 µmol/L within 48 h. We considered only 1 AKI episode per hospitalization; however, a patient could have recurrent AKI episodes if hospitalized multiple times during the follow-up period.

**Table 1:** Definition and classification of AKI.

Acute kidney Injury	Definition	End of follow-up
Ascertainment	Detection based on following criteria:	31st December each calendar year, death or migration
	Serum/plasma-Cr ≥ 1.5 times the baseline value (BV), defined as the median outpatient serum/plasma creatinine measurements 1-365 days prior hospital admission.	
	Serum/plasma-Cr ≥ 26.5 µmol/L higher than creatinine measurement the preceding 2 days (48 h) during hospital stay.	
	Acute kidney injury ICD-10 code N17 (at hospital discharge, any position).	
	Need of acute dialysis register code DR015 and DR023.	
Severity categorization into 3 stages:	Stage 1: Creatinine elevation 1.5–1.99 times higher than BV or increase of ≥ 26.5 µmol/L within 48 h (and ≤ 353.6 µmol/L).	
	Stage 2: Creatinine elevation 2.0-2.99 times higher than BV.	
	Stage 3: Creatinine elevation ≥ 3 times higher than BV. Creatinine increase of ≥ 353.6 µmol/L within 48 h.	

### Statistical analyses

Categorical variables were summarized as frequencies and percentages; continuous variables as medians with interquartile ranges (IQRs). We quantified the number of AKI events per year and calculated AKI incidence rates (IR) through Poisson regression. To explore trends over time we then calculated incidence rate ratios (IRR) with year 2017 as referent, applying robust standard errors to account for the correlation between repeated person-year observations in the same individual.

We explored IRR of AKI about hypothesized high-risk subgroups. We did this at each calendar year but presented the most recent year in our main results, the remaining in the supplementary materials. Since we were interested in evaluating the statistical association between each risk factor and AKI rather than the causality, we abstained from adjusting for potential confounders.

Finally, to assess the relative contribution of each risk factor to AKI occurrence, we performed deviance analysis based on a multivariable Poisson regression model including all predictors^30^. Unlike adjusted incidence rate ratios, which quantify the strength of association for each risk factor independently of its prevalence or distribution, deviance is the Poisson-regression analogue of the residual sum of squares and can be partitioned across covariates to yield a common, directly comparable metric of their contribution to explained variation in AKI occurrence. We therefore considered this approach more appropriate than inspection of adjusted IRRs for the specific purpose of ranking risk factors by their population-level importance, rather than estimating their individual effect sizes.

Statistical analysis was performed using RStudio statistical software (version 2025.09.2).

## Results

### Population characteristics

There has been a relatively stable population in the Region of Stockholm during 2017 to 2021, from 1.942.000 registered citizens in 2017 to 1.890.000 in 2021. The characteristics are summarized in Table 2. Demographics and the proportion of patients with comorbid conditions have been largely consistent across years. In 2021, the median age of the Stockholm population was 46 years (IQR 33.0–61.0), 51% were female, and the median eGFR (amongst those tested) was 90 ml/min/1.73 m^2^ (IQR 76.0-103.0). Approximately 30% of the total population had at least one creatinine test measured the preceding year. Only 12% of the population had a urine albumin sample tested in the year prior (either through dipstick or urine albumin-creatinine ratio, UACR), and the majority had A1. Hypertension was common, in nearly 20% of the citizens. As many as 0.5% had a previous clinical diagnosis of AKI.

**Table 2:** Baseline characteristics of the Stockholm population per calendar year Abbreviations: COPD; Chronic obstructive pulmonary disease. HIV; Human immunodeficiency virus, AIDS; Acquired immunodeficiency syndrome.

	2017	2018	2019	2020	2021
	*N* = 1,942,303	*N* = 1,933,005	*N* = 1,920,329	*N* = 1,908,610	*N* = 1,890,312
Age, years (median, IQR)	44 (31, 60)	44 (31, 60)	45 (31, 61)	46 (32, 61)	46 (33, 61)
Female (%)	992,290 (51)	986,289 (51)	979,321 (51)	972,975 (51)	962,996 (51)
**Education level (%)**					
Compulsory school	238,346 (12)	245,443 (13)	260,546 (14)	275,812 (15)	276,343 (15)
Secondary school	702,966 (36)	697,751 (36)	681,798 (36)	666,184 (35)	649,791 (34)
University	868,051 (45)	853,461 (44)	840,043 (44)	828,025 (43)	814,470 (43)
Missing	132,940 (7)	136,350 (7)	137,942 (7)	138,589 (7)	149,708 (8)
eGFR, ml/min/1.73 m^2^ (median, IQR)	88 (74, 101)	87 (73, 100)	87 (73, 100)	87 (73, 100)	90 (76, 103)
**eGFR stage (%)**					
<15 ml/min/1.73 m^2^	470 (0.0)	490 (0.0)	478 (0.0)	465 (0.0)	427 (0.0)
15–29 ml/min/1.73 m^2^	3,859 (0.2)	4,006 (0.2)	3,875 (0.2)	3,848 (0.2)	3,412 (0.2)
30–44 ml/min/1.73 m^2^	15,251 (0.8)	16,432 (0.9)	15,717 (0.8)	16,231 (0.9)	13,788 (0.7)
45–59 ml/min/1.73 m^2^	40,895 (2.1)	45,255 (2.3)	43,357 (2.3)	46,117 (2.4)	36,424 (1.9)
60–89 ml/min/1.73 m^2^	259,351 (13)	276,204 (14)	255,548 (13)	281,609 (15)	235,556 (13)
90–104 ml/min/1.73 m^2^	153,809 (8)	152,432 (8)	141,141 (7)	160,759 (8)	156,565(8)
>105 ml/min/1.73 m^2^	112,989 (6)	106,874 (6)	99,513 (5)	113,765 (6)	121,945 (7)
Missing	1,355,679 (70)	1,331,312 (69)	1,360,700 (71)	1,285,816 (67)	1,322,195 (70)
**Albuminuria (%)**					
<30 mg/g	164,063 (8)	173,921 (9)	169,641 (9)	201,866 (11)	194,768 (10)
30–300 mg/g	37,017 (2)	39,337 (2)	35,054 (2)	39,204 (2)	33,488 (2)
>300 mg/g	22,798 (1)	22,600 (1)	21,351 (1)	23,043 (1)	21,493 (1)
Missing	1,718,425(89)	1,697,147 (88)	1,694,283 (88)	1,644,497 (86)	1,640,563 (87)
Diabetes mellitus (%)	101,969 (5.2)	106,421 (5.5)	111,066 (5.8)	115,708 (6.1)	118,098 (6.2)
**HbA1c (in diabetics)**					
<6%	11,658 (11)	13,056 (12)	12,199 (11)	15,109 (13)	14,788 (13)
6–6.9%	31,588 (31)	32,494 (31)	33,354 (30)	37,330 (32)	35,529 (30)
7–7.9%	19,858 (19)	20,138 (19)	20,323 (18)	22,295 (19)	20,451 (17)
8–8.9%	10,208 (10)	10,333 (10)	9,811 (9)	10,679 (9)	9,763 (8)
≥ 9%	7,749 (8)	7,740 (7)	7,506 (7)	8,166 (7)	7,819 (7)
Missing	20,908 (21)	22,660 (21)	27,873 (25)	22,129 (19)	29,748 (25)
COVID-19 infection (%)	–	–	–	61,185 (3.2)	70,319 (3.7)
Hypertension (%)	330,433 (17)	341,216 (18)	351,624 (18)	363,722 (19)	370,179 (20)
Kidney transplant recipients (%)	528 (< 0.1)	544 (0.0)	543 (0.0)	553 (0.0)	546 (0.0)
Acute coronary syndrome (%)	34,920 (1.8)	35,517 (1.8)	36,119 (1.9)	36,795 (1.9)	36,437 (1.9)
Other ischemic heart disease (%)	69,469 (4)	69,697 (4)	69,959 (4)	70,532 (4)	68,426 (4)
Heart failure (%)	39,400 (2)	40,057 (2)	40,653 (2)	41,860 (2)	40,677 (2)
Heart valvular disorder (%)	17,144 (0.9)	18,283 (0.9)	19,867 (1.0)	21,648 (1.1)	21,911 (1.2)
Atrial fibrillation/flutter (%)	60,516 (3)	62,368 (3)	64,164 (3)	66,243 (4)	65,961 (4)
Other arrhythmias (%)	64,342 (3)	66,899 (4)	69,519 (4)	72,550 (4)	72,966 (4)
Stroke (%)	36,566 (2)	37,147 (2)	37,697 (2)	38,409 (2)	37,944 (2)
Other cerebrovascular disease (%)	44,092 (2)	45,564 (2)	46,711 (2)	48,343 (3)	47,887 (3)
Peripheral vascular disease (%)	20,434 (1.1)	21,020 (1.1)	21,696 (1.1)	22,303 (1.2)	21,934 (1.2)
Renal artery stenosis (%)	313 (< 0.1)	325 (0.0)	338 (0.0)	343 (0.0)	348 (0.0)
COPD (%)	41,841 (2)	43,320 (2)	44,554 (2)	45,815 (2)	45,019 (2)
Other lung disease (%)	214,276 (11)	225,785 (12)	238,209 (12)	250,223 (13)	258,927 (14)
Venous thrombo-embolism (%)	46,082 (2)	47,781 (3)	49,738 (3)	51,996 (3)	52,988 (3)
Cancer without metastasis (%)	103,541 (5)	108,078 (6)	111,649 (6)	115,777 (6)	114,210 (6)
Cancer with metastasis (%)	16,644 (0.9)	17,749 (0.9)	18,707 (1.0)	19,905 (1.0)	19,293 (1.0)
Multiple myeloma (%)	1,677 (< 0.1)	1,744 (0.1)	1,768 (0.1)	1,835 (0.1)	1,798 (0.1)
Liver disease (%)	28,900 (1.5)	30,426 (1.6)	31,986 (1.7)	33,516 (1.8)	34,050 (1.8)
Inflammatory bowel disease (%)	20,289 (1.0)	21,064 (1.1)	22,079 (1.1)	23,150 (1.2)	23,215 (1.2)
Rheumatic disease	41,926 (2)	43,882 (2)	45,734 (2)	47,493 (3)	47,464 (3)
Pancreatitis (%)	9,931 (0.5)	10,452 (0.5)	10,959 (0.6)	11,530 (0.6)	11,831 (0.6)
Fracture (%)	330,784 (17)	346,809 (18)	363,147 (19)	380,222 (20)	388,762 (21)
Psychiatric disorder (%)	582,640 (30)	617,957 (32)	653,602 (34)	690,357 (36)	717,930 (38)
HIV/AIDS (%)	3,504 (0.2)	3,615 (0.2)	3,703 (0.2)	3,822 (0.2)	3,763 (0.2)
Dementia (%)	18,345 (0.9)	18,618 (1.0)	18,868 (1.0)	19,361 (1.0)	16,902 (0.9)
History of AKI (%)	7,401 (0.4)	8,449 (0.4)	9,568 (0.5)	10,924 (0.6)	12,002 (0.6)
Coronary revascularization (%)	29,408 (1.5)	30,362 (1.6)	31,082 (1.6)	31,806 (1.7)	31,875 (1.7)
Other cardiac operation (%)	28,076 (1.4)	28,537 (1.5)	29,432 (1.5)	30,473 (1.6)	31,030 (1.6)
Colectomy (%)	1,341 (< 0.1)	1,373 (0.1)	1,427 (0.1)	1,462 (0.1)	1,529 (0.1)

Abbreviations: COPD; Chronic obstructive pulmonary disease. HIV; Human immunodeficiency virus, AIDS; Acquired immunodeficiency syndrome

### AKI incidence and time trends

Every year, 0.6–0.7% of the total population of Stockholm experienced AKI (Table 3). This corresponds to incidence rates that range between 71 and 95 cases per 10.000 person years. The majority of AKIs (≈ 90%) were identified via transient creatinine elevations and were not recognized with a clinical diagnosis. The majority (about 68–69%) of AKIs were classified as mild (KDIGO stage I severity). A clinically relevant proportion of hospital admissions was attributed to AKI or was complicated with an AKI, being present in between 5.8% and 7.8% of all hospitalizations in the region.

**Table 3:** Incidence, incidence rate and stages of acute kidney injury across calendar years.

	2017	2018	2019	2020	2021
Population of Stockholm (*n*)	1 942 303	1 933 005	1 920 329	1 908 610	1 890 312
Unique individuals experiencing AKI (n)	11 644	10 892	11 588	12 424	13 591
**Source of AKI identification (non-exclusive)**					
Creatinine elevations	10 689	9746	10 307	11 009	12 025
ICD-10 diagnosis	2960	3170	3632	4381	5072
Provision of acute dialysis	92	105	121	162	146
Annual population incidence	0.60%	0.56%	0.60%	0.65%	0.72%
Total number of AKI events^1^	14 421	13 402	14 422	15 501	17 638
Severity Stage I^2^	8797 (69.0%)	7865 (68.6%)	8436 (68.8%)	8913 (69.0%)	9901 (68.2%)
Severy Stage II^2^	2503 (19.6%)	2277 (19.9%)	2458 (20.1%)	2442 (18.9%)	2900 (20.0%)
Severity Stage III^3^	1445 (11.3%)	1328 (11.6%)	1362 (11.1%)	1568 (12.1%)	1716 (11.8%)
Unclassified severity^4^	1676	1932	2166	2578	3121
AKI Incidence rate, per 10.000 years^1^	75.7 (74.4–76.9)	70.7 (69.5–71.9)	76.6 (75.3–77.8)	82.7 (81.4–84.0)	95.3 (93.9–96.7)
Incidence rate ratio (95% CI)	Ref.	0.93 (0.91–0.96)	1.01 (0.99–1.04)	1.09 (1.07–1.12)	1.26 (1.23–1.29)
Total number of hospitalizations in the region	238 119	232 936	234 313	218 376	227 125
Proportion of hospitalizations with AKI (%)	6.1%	5.8%	6.2%	7.1%	7.8%

^1^Including recurrent events.

^2^Derived from laboratory measurements.

^3^Derived from laboratory measurements and/or acute dialysis provision code.

^4^Derived from ICD-10 registry.

In time trend analysis, there appears to be a trend towards increasing AKI occurrence. The incidence rate increased from 75.7 (95% CI 74.4–76.9) events per 10.000 years in 2017 to 95.3 (95% CI 93.9–96.7) events in 2021. This corresponds to a relative increase of 26% (IRR, 1.26; 95% CI 1.23–1.29) (Table 3).

### Populations at risk of AKI

**Fig. 1:**
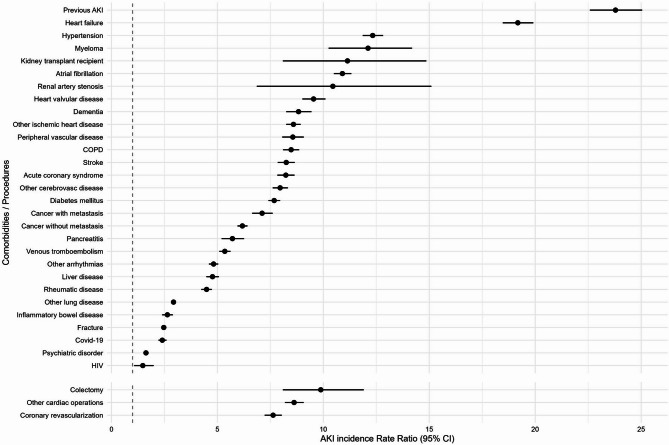
Forest plot depicting univariable incidence rate ratios (IRR) of AKI for selected comorbid conditions and undertaken procedures in the Stockholm population of year 2021. Incidence rate ratios comparing AKI rates among participants with (vs. without) the given comorbid condition. Abbreviations: COPD; Chronic obstructive pulmonary disease. HIV; Human immunodeficiency virus.

Among key comorbid conditions (Fig. 1), a history of AKI and heart failure were strongly associated with AKI occurrence, with IRRs of 24 (95% CI 22.6–25.0) and 19 (95% CI 18.5–20) fold higher, respectively. Other comorbidities with high risk estimates were hypertension (IRR 12, 95% CI 11.9–12.8), multiple myeloma (IRR 12, 95% CI 10.3–14) and kidney transplant recipients (IRR 11, 95% CI 8–15). The risk estimate for patients with diabetes mellitus (vs. non-diabetics) was IRR 7.7, 95% CI 7.4-8.0. Notably, people with a history of colectomy had almost a 10-fold higher risk of AKI (vs. those without, IRR 9.88, 95% CI 8.1–12.0). Similar risk patterns were observed in the years preceding 2021 (Supplementary Table S2). A positive test of COVID-19 infection in 2020 was associated with almost 6-fold higher risk of AKI (IRR 5.7, CI 5.2–6.2). In the following year (2021), the risk estimate was reduced to a 2.5-fold higher risk (IRR 2.4, CI 2.2–2.6).

**Fig. 2:**
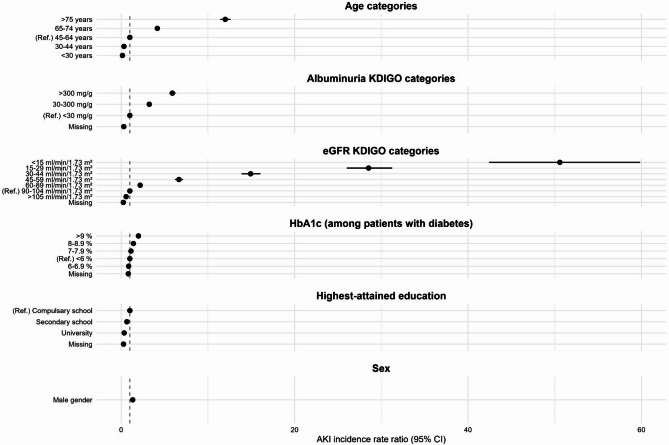
Forest plot depicting univariable incidence rate ratios (IRR) of AKI for selected demographics and laboratory-based variables in the Stockholm population of year 2021. Incidence rate ratios of demographics and laboratory-based variables comparing AKI rates across different subgroups. Each variable with its own reference group (IRR=1). *Note*: The X-axis scale differs between Figs. 1 and 2. Abbreviations: Ref; Reference. A1;< 30 mg/g. A2;30-300 mg/g. A3;>300 mg/g. eGFR; estimated glomerular infiltration rate

Among sociodemographic characteristics (Fig. 2), older adults were more likely to experience AKI: Compared with middle-aged (age 45–64 years), those aged ≥ 75 years had a 12-fold risk of AKI (IRR 12, 95% CI 11.4–12.6). Higher attained educational level was associated with a lower risk of AKI: people with a university degree had an IRR of 0.35 (95% CI 0.33–0.36) compared to people who achieved compulsory school education. Men had overall a 33% (IRR 1.33; 95% CI 1.28–1.38) higher risk of AKI compared to women. These risk associations were consistent across all years. (Supplementary Table S3).

Lower categories of eGFR, more severe categories of albuminuria and an elevated HbA1c (among people with diabetes) were all associated with higher rates of AKI in a graded fashion (Fig. 2). Participants with an eGFR of 15–29 ml/min/1.73 m^2^ had 29 times higher risk of AKI (95% CI 26–31) compared with participants with eGFR > 90 ml/min/1.73 m^2^. Participants with KDIGO A3 category (albuminuria > 300 mg/g) had nearly six-fold higher risk of AKI (IRR 5.9, 95% CI 5.6–6.3) compared to participants with normoalbuminuria (KDIGO category A1). Similarly, HbA1c ≥ 9% had twice as high risk of AKI (IRR 1.99, 95% CI 1.77–2.24). Not all individuals had these tests taken, and participants missing any of these laboratory tests were at lower risk of AKI, probably reflecting their healthier condition (i.e. there was no suspicion of kidney damage that would prompt testing for creatinine or albuminuria). These associations were consistent across years (Supplementary Table S3).

**Fig. 3:**
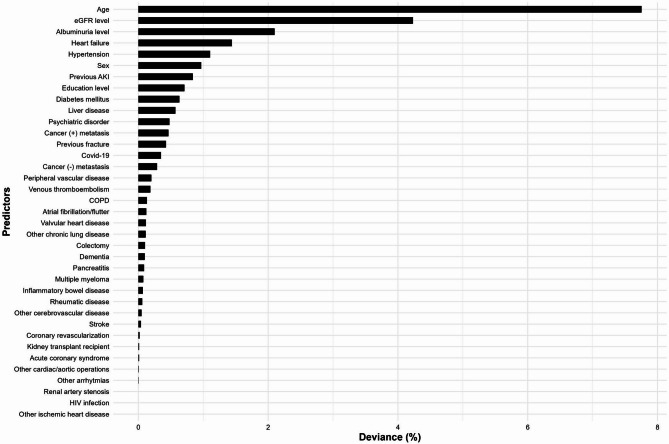
Relative deviance contribution of comorbid conditions to the risk of AKI in the population of Stockholm, year 2021. Deviance analysis based on Poisson regression model. For each covariate, the reduction in model deviance was calculated stepwise and expressed as a proportion of total explained deviance.Abbreviations: eGFR; estimated glomerular infiltration rate. COPD; Chronic obstructive pulmonary disease. HIV; Human immunodeficiency virus

In deviance analysis (Fig. 3), the conditions that contributed the most in multivariable models to explain the occurrence of AKI were old age, followed by low eGFR and albuminuria. Other covariates that had a significant impact on predicting AKI were heart failure and hypertension. These associations were consistent across years (Supplementary Table S4). Among 37 risk factors, COVID-19 infection was ranked the 14th most important contributor in both year 2020 and 2021.

## Discussion

In this large study covering the complete population of the region of Stockholm, Sweden, we made the following observations: (a) AKI is common, occurring in 6–8% in all hospital admissions; (b) there appears to be a trend towards increasing AKI incidence in the most recent years; (c) populations at risk of AKI are largely consistent over the years, and convey principally people with old age, men, uncontrolled diabetes, history of AKI, hypertension or heart failure. Even mild signs of kidney dysfunction (eGFR) or kidney damage (albuminuria) associated with higher AKI rates. Collectively, this analysis comprehensively quantifies the burden of AKI in an entire region and identifies populations that may benefit from preventative and/or monitoring programs.

Our comprehensive and contemporary evaluation in a complete health system complements previous reports on the burden of AKI. Approximately 0.6–0.7% of adults in Stockholm experienced AKI that led to hospitalization or occurred during the hospitalization. Our estimates are relatively comparable with a report from Canada, Denmark, and the UK^20^, where the AKI incidence in-hospital was reported between 89 and 100 per 10,000 person-years during 2011–2014 (in our study 71–95 AKI events per 10,000 person years). Direct comparison is not straightforward given differences in the underlying data granularity, definitions, periods and diagnosis or testing rates between countries. In an older meta-analysis from 2015 (primarily including North America general population cohorts) AKI was estimated to affect 1.3% of the general population^15^. In a U.S. Veterans study^21^ the AKI incidence was reported as 1.9% annually. We do not think there are true differences in AKI incidence across studies, but instead they are an artifact of the characteristics of the populations included. Our study evaluated the totality of the adult population of Stockholm, whereas in the U.S. Veterans study, the population was primarily male of older age.

We also report that 6–8% of hospital admissions are related with AKI. This estimate is again lower than previous reports^1,31^ and most likely reflects our complete-case evaluation. In contrast, many previous studies have focused on specific patient care context. Among patients who underwent coronary artery bypass grafting (CABG), 13% of patients developed postoperative AKI^32^. Following various major surgical procedures, the incidence was 18%^33^.

An intriguing finding of our study is a concerning rising AKI incidence from 2017 to 2021, the most recent period available to us. The drivers of this increase are unclear and contrast with earlier reports of stable AKI rates over time^20–22,34^. One possibility is enhanced creatinine testing and heightened clinical awareness; however, the KDIGO criteria were introduced in 2012, well before our study period, making this explanation incomplete. Another potential contributor is the COVID-19 pandemic, as early reports estimated AKI incidence among hospitalized COVID-19 patients at approximately 28%^35^. Nevertheless, AKI rates following COVID-19 declined substantially from 2020 to 2021, whereas we observed the highest AKI incidence in 2021, suggesting that the pandemic alone is unlikely to account for the observed trend. What is behind the risk attenuation in COVID-19 patients is not clear. We speculate it may relate to depletion of the susceptibles first, mass vaccination campaigns (with subsequent hospital admissions of milder severity), and better in-hospital care. We recognized that lack of data beyond 2021 is a limitation of our study, and confirmation -as well as expansion- of these trends in more recent years and across other healthcare systems is warranted.

Characterizing AKI risk in different high-risk groups highlights the need for AKI-preventive strategies in these conditions. Beyond the clear importance of underlying kidney function, albuminuria, and previous history of AKI, our multivariate risk analyses confirm the diversity of the risk factors involved in AKI^15,16^, which include older age, hypertension, heart failure, multiple myeloma, and kidney transplantation, among others. In patients with diabetes, poor glycemic control was also an important predictor of AKI. Our results contribute with new knowledge and may serve as a discussion point for updated policies regarding kidney function screening as part of risk assessment and planning to prevent AKI. Preventive measures may include avoidance of AKI-inducing drugs, haemodynamic optimization and patient education on temporarily withholding selected drugs (e.g., renin–angiotensin system inhibitors) during episodes of dehydration, vomiting, or diarrhea (“sick day rules”^29,36^. Finally, we observed that lower educational attainment was associated with a higher risk of AKI. This is consistent with findings from a prior cohort study^37^, and may point to a population subgroup in which enhanced screening, education, and awareness of AKI triggers could be particularly beneficial^38^. Because identified risk predictors are generally in line with preceding publications from distant geographical locations^20–22,31,34^, we believe our findings may be generalizable to other settings.

Strengths of our analysis include complete regional capture of healthcare utilization within a universal healthcare system and inclusion of the entire adult population, thereby minimizing selection bias common to prior studies. Moreover, reliance on ICD codes alone is known to substantially underestimate AKI incidence^21,39^. Accordingly, we view our multi-source AKI ascertainment—integrating diagnoses, procedures, and laboratory abnormalities—as a major strength. Key limitations include the lack of data after 2021, when severe COVID-19 infections rates subsequently substantially decreased and novel medications with potential to reduce AKI incidence (e.g. SGLT2-I^40^ became the new standard of care. We did neither evaluate community-managed AKI and our observational design precludes drawing causal inferences from crude incidence rates. However, we deliberately reported crude rates, as we expect clinicians to interpret these data pragmatically—namely, to gauge AKI risk associated with specific conditions in real-world patients rather than adjusted estimates conditional on multiple covariates. Finally, we acknowledge that serum creatinine and albuminuria measurements may have been ordered for a variety of clinical indications beyond the assessment of kidney function. We sought to minimize this potential source of bias by restricting these measurements to the outpatient setting; however, residual confounding cannot be excluded.

To conclude, this region-wide analysis quantifies the contemporary burden of AKI and identifies high-risk populations that may benefit from targeted prevention and monitoring strategies.

## Supplementary Information

Below is the link to the electronic supplementary material.

Supplementary Material 1

## Data Availability

The data underlying this article cannot be shared publicly due to the privacy of individuals who participated in the study. The data may be shared on reasonable request for academic research collaborations that fulfil GDPR as well as national and institutional ethics regulations and standards by contacting Prof. Juan-Jesus Carrero (juan.jesus.carrero@ki.se).
