# Supplementary material for: Incidence, time trends and predictors of acute kidney injury in the region of Stockholm, Sweden
Source: Sci Rep. 2026 Aug 15;16:25525. doi: 10.1038/s41598-026-67110-y (PMC13477473; doi:10.1038/s41598-026-67110-y)
Supplement: Supplementary file 1 [file 41598_2026_67110_MOESM1_ESM.docx]

**Supplementary**

**Table S1. Definition of covariates.**

| **Demographics (CED):** |  |
| --- | --- |
| Age | Age at initiation |
| Sex | Registered sex |
| Calendar year | Index year of cohort entry |
| Education | 1 = Compulsory school  2 = Secondary school  3 = University  4 = Missing |
| **Labs (previous 12 months, CED):** |  |
| eGFR_12 months | Average during previous 12 months before CED (incl. missing category) |
| CKD stage_12 months | G1-G5, based on average eGFR during previous 12 months before CED (incl. missing category) |
| Albuminuria | A1-A3, based on several sources (urine dipstick, UACR etc.) (incl. missing category). If ≥ 1 take the most severe |
| HbA1c_12 months (only if diabetes diagnosis below?) | Average HbA1c during previous 12 months before CED (incl. missing category) |
| **Comorbidities (∞, CED if not stated else):** | **ICD-10 code** |
| Hypertension | I10-15 |
| Diabetes mellitus | E100-E108, E109, E110-E116, E117, E118, E119, E130-E136, E137, E138, E139, E140-E148, E149, E160, E161, E162, G990, G590, G632, H280, H358, H360, M142, M146 |
| Kidney transplant recipient | Z94.0 |
| Acute coronary syndrome | I200, I21, I22 |
| Other ischemic heart disease | I201, I208, I209, I24, I25 |
| Heart failure | I110, I130, I132, I50 |
| Valvular disorder | I34, I35, I36, I37 |
| Stroke | I60, I61, I62, I63, I64 |
| Other cerebrovascular disease | I65, I66, I67, I68, I69, G450, G451, G452, G453, G458, G459, G46 |
| Atrial fibrillation/flutter | I48 |
| Other arrhythmias | I44, I45, I46, I47, I49 |
| Peripheral vascular disease | I70.2, I72.1, I72.4, I73 |
| Renal artery stenosis/atherosclerosis | I70.1 |
| Chronic obstructive pulmonary disease (COPD) | J44 |
| Other chronic lung disease | I270, I272, I278, I279, J40, J41, J42, J43, J44, J45, J46, J47, J60, J61, J62, J63, J64, J65, J66, J67, J68, J69, J70, J84, J92, J96, J982, J983 |
| Venous thromboembolism | I26, I801, I802, I803, I808, I809, I81, I820, I822, I823, I828, I829 |
| Cancer **(previous 12 months, CED)**  Without metastasis (non-melanoma skin cancer excluded)      With metastasis  Multiple myeloma | C01-C26, C30-C34, C37-C41, C43, C45-C58, C60-C76, C81-C86, C88, C91-C97  C77, C78, C79  C90 |
| Liver disease | B18, I850, I859, I982, K70, K71, K72, K73, K74, K75, K76, K77 |
| Inflammatory bowel disease | K50, K51 |
| Rheumatic disease | M05, M06, M07, M08, M09, M30, M31, M32, M33, M34, M351, M353, M45 |
| Psychiatric disorder | F04, F05, F06, F07, F09, F10, F2[0-9], F3[0-9], F4[0-9], F5[0-9], F6[0-9], F7[0-9], F8[0-9], F9[0-9] |
| Pancreatitis | K85, K860, K861 |
| Fracture | S020, S021, S022, S023, S024, S026, S027, S028, S029, S12, S22, S32, S42, S52, S62, S72, S82, S92, T02, T08, T10, T12, M484, M485, M843 |
| HIV/AIDS | B20-B24 |
| Dementia | F00, F01, F02, F03 |
| Previous AKI | N17 |
|  |  |
| **Procedures** | **KVÅ code** |
| Cardiac revascularization procedures | FNG00, FNG02, FNG05, FNA, FNB, FNC, FND, FNE, FNF, FNH, FNJ, FNK, FNW |
| Other cardiac/aortic/valve operation | F codes (except the ones above) |
| Colectomy | JFH00, JFH01, JFH10, JFH11, JFH20,  JFH30, JFH31, JFH33, JFH40, JFH96 |

**Table S2. Univariable incidence rate ratios (IRR) of AKI for selected comorbid conditions and undertaken procedures in the Stockholm population, 2017-2021.**

|  | **2017** | **2018** | **2019** | **2020** | **2021** |
| --- | --- | --- | --- | --- | --- |
| **Previous AKI** | 26.7 (25.0-28.5) | 27.4 (25.7-29.2) | 25.6 (24.1-27.2) | 24.1 (22.8-25.5) | 23.8 (22.6-25.0) |
| **Heart failure** | 20.7 (19.9-21.6) | 20.8 (19.9-21.6) | 20.4 (19.6-21.2) | 19.2 (18.5-20.0) | 19.2 (18.5-19.9) |
| **Hypertension** | 12.2 (11.7-12.7) | 12.7 (12.2-13.2) | 13.4 (12.8-13.9) | 12.2 (11.8-12.8) | 12.3 (11.9-12.8) |
| **Myeloma** | 13.5 (11.3-16.0) | 15.2 (12.8-17.9) | 12.9 (10.8-15.3) | 11.7 (9.8-13.9) | 12.1 (10.2-14.2) |
| **Kidney transplant recipient** | 17.6 (13.3-22.9) | 14.8 (10.8-19.7) | 14.1 (10.3-18.7) | 11.4 (8.2-15.3) | 11.1 (8.1-14.9) |
| **Atrial fibrillation** | 11.8 (11.3-12.3) | 12.0 (11.5-12.5) | 11.7 (11.2-12.2) | 10.8 (10.4-11.2) | 10.9 (10.5-11.3) |
| **Renal artery stenosis** | 10.0 (6.0-15.3) | 11.3 (7.0-17.0) | 9.6 (5.9-14.6) | 13.8 (9.4-19.5) | 10.4 (6.9-15.1) |
| **Heart valvular disease** | 10.9 (10.2-11.6) | 10.4 (9.8-11.2) | 10.7 (10.1-11.4) | 9.6 (9.0-10.2) | 9.5 (9.0-10.1) |
| **Dementia** | 9.4 (8.8-10.1) | 9.9 (9.2-10.6) | 8.7 (8.1-9.3) | 9.3 (8.7-9.9) | 8.8 (8.2-9.4) |
| **Other ischemic heart disease** | 9.3 (8.9-9.7) | 9.1 (8.7-9.5) | 9.5 (9.1-9.9) | 8.7 (8.3-9.0) | 8.6 (8.2-8.9) |
| **Peripheral vascular disease** | 10.7 (10.1-11.4) | 10.1 (9.4-10.7) | 10.1 (9.5-10.7) | 9.0 (8.4-9.5) | 8.6 (8.1-9.1) |
| **COPD** | 9.7 (9.2-10.1) | 9.2 (8.8-9.7) | 9.1 (8.7-9.6) | 8.2 (7.8-8.6) | 8.5 (8.1-8.9) |
| **Stroke** | 8.7 (8.2-9.2) | 9.3 (8.8-9.8) | 8.9 (8.4-9.3) | 8.6 (8.1-9.0) | 8.2 (7.8-8.7) |
| **Acute coronary syndrome** | 8.9 (8.5-9.4) | 8.8 (8.4-9.3) | 9.1 (8.6-9.5) | 8.5 (8.1-8.9) | 8.2 (7.8-8.6) |
| **Other cerebrovasc disease** | 8.1 (7.7-8.5) | 8.8 (8.3-9.2) | 8.5 (8.1-8.9) | 8.2 (7.8-8.6) | 8.0 (7.6-8.3) |
| **Diabetes mellitus** | 8.2 (7.8-8.5) | 8.2 (7.9-8.6) | 8.0 (7.7-8.3) | 8.0 (7.7-8.3) | 7.7 (7.4-8.0) |
| **Cancer with metastasis** | 8.8 (8.2-9.5) | 8.6 (7.9-9.2) | 8.1 (7.6-8.7) | 7.6 (7.1-8.1) | 7.1 (6.6-7.6) |
| **Cancer without metastasis** | 7.0 (6.7-7.3) | 6.7 (6.4-7.0) | 6.7 (6.4-7.0) | 6.3 (6.0-6.5) | 6.2 (6.0-6.4) |
| **Pancreatitis** | 5.8 (5.2-6.4) | 5.9 (5.3-6.6) | 6.0 (5.4-6.6) | 5.5 (5.0-6.1) | 5.7 (5.2-6.3) |
| **Venous tromboembolism** | 5.6 (5.3-5.9) | 5.8 (5.5-6.1) | 5.6 (5.3-5.9) | 5.4 (5.1-5.7) | 5.3 (5.1-5.6) |
| **Other arrhythmias** | 5.2 (5.0-5.5) | 5.2 (4.9-5.5) | 5.2 (5.0-5.5) | 4.8 (4.6-5.1) | 4.8 (4.6-5.0) |
| **Liver disease** | 5.2 (4.8-5.6) | 5.5 (5.1-5.9) | 5.3 (5.0-5.7) | 4.8 (4.4-5.1) | 4.8 (4.5-5.1) |
| **Rheumatic disease** | 5.2 (4.9-5.5) | 4.8 (4.6-5.2) | 4.8 (4.6-5.1) | 4.6 (4.3-4.9) | 4.5 (4.2-4.7) |
| **Other lung disease** | 3.4 (3.3-3.5) | 3.4 (3.2-3.5) | 3.2 (3.1-3.3) | 2.9 (2.8-3.0) | 2.9 (2.8-3.0) |
| **Inflammatory bowel disease** | 2.7 (2.4-3.0) | 2.7 (2.5-3.1) | 2.8 (2.5-3.1) | 2.5 (2.3-2.8) | 2.6 (2.4-2.9) |
| **Fracture** | 2.8 (2.7-2.9) | 2.7 (2.6-2.8) | 2.6 (2.5-2.7) | 2.5 (2.4-2.6) | 2.5 (2.4-2.6) |
| **Covid-19 infection** | - | - | - | 5.7 (5.2-6.2) | 2.4 (2.2-2.6) |
| **Psychiatric disorder** | 2.0 (2.0-2.1) | 2.0 (1.9-2.1) | 1.8 (1.7-1.9) | 1.7 (1.6-1.7) | 1.6 (1.6-1.7) |
| **HIV** | 1.8 (1.2-2.4) | 1.6 (1.1-2.2) | 1.6 (1.1-2.2) | 1.9 (1.4-2.5) | 1.5 (1.1-2.0) |
| **Colectomy** | 8.9 (7.0-11.2) | 8.9 (7.0-11.3) | 9.3 (7.4-11.5) | 8.1 (6.4-10.1) | 9.9 (8.1-11.9) |
| **Other cardiac operations** | 9.1 (8.5-9.6) | 9.0 (8.5-9.5) | 9.0 (8.5-9.5) | 8.5 (8.0-9.0) | 8.6 (8.2-9.1) |
| **Coronary revascularization** | 7.5 (7.0-7.9) | 7.8 (7.3-8.2) | 8.0 (7.5-8.4) | 7.9 (7.4-8.3) | 7.6 (7.2-8.0) |

**Table S3. Univariable incidence rate ratios (IRR) of AKI for selected demographics and laboratory-based variables in the Stockholm population, 2017-2021.**

|  | **2017** | **2018** | **2019** | **2020** | **2021** |
| --- | --- | --- | --- | --- | --- |
| **Age categories** |  |  |  |  |  |
| ≥ 75 | 11.2 (10.7-11.8) | 11.8 (11.2- 12.5) | 11.7 (11.1-12.3) | 10.8 (10.3-11.4) | 12.0 (11.4-12.6) |
| 65-74 | 3.8 (3.6-4.0) | 4.1 (3.8-4.3) | 4.0 (3.8-4.3) | 3.8 (3.6-4.0) | 4.2 (4.0-4.4) |
| 45-64 (ref.) | 1.0 | 1.0 | 1.0 | 1.0 | 1.0 |
| 30-44 | 0.25 (0.23-0.28) | 0.26 (0.23-0.29) | 0.27 (0.24-0.30) | 0.27 (0.25-0.30) | 0.32 (0.29-0.35) |
| <30 | 0.14 (0.12-0.16) | 0.14 (0.12-0.16) | 0.14 (0.12-0.17) | 0.14 (0.12-0.16) | 0.15 (0.13-0.18) |
|  |  |  |  |  |  |
| **Albuminuria KDIGO categories** |  |  |  |  |  |
| A3 | 5.9 (5.5-6.3) | 6.1 (5.7-6.5) | 5.8 (5.5-6.2) | 6.1 (5.80-6.5) | 5.9 (5.6-6.3) |
| A2 | 3.2 (3.0-3.4) | 3.1 (2.9-3.3) | 3.1 (2.9-3.4) | 3.1 (2.9-3.3) | 3.2 (3.1-3.4) |
| A1 | 1.0 | 1.0 | 1.0 | 1.0 | 1.0 |
| Missing | 0.29 (0.28-0.30) | 0.28 (0.27-0.30) | 0.28 (0.27-0.30) | 0.28 (0.26-0.29) | 0.30 (0.28-0.31) |
|  |  |  |  |  |  |
| **Higher-attained education** |  |  |  |  |  |
| Compulsory school | 1.0 | 1.0 | 1.0 | 1.0 | 1.0 |
| Secondary school | 0.46 (0.45-0.49) | 0.49 (0.47-0.51) | 0.55 (0.52- 0.57) | 0.60 (0.58-0.63) | 0.66 (0.63-0.68) |
| University | 0.23 (0.22-0.24) | 0.24 (0.23-0.25) | 0.28 (0.26-0.29) | 0.32 (0.31-0.34) | 0.35 (0.33-0.36) |
| Missing | 0.18 (0.16-0.20) | 0.17 (0.15-0.19) | 0.21 (0.19-0.23) | 0.26 (0.24-0.29) | 0.27 (0.25-0.30) |
|  |  |  |  |  |  |
| **Baseline kidney function KDIGO (eGFR)** |  |  |  |  |  |
| <15 | 57.2 (48.2-67.0 | 51.8 (43.0-61.9) | 51.2 (42.6-61.0) | 59.0 (50.0-70.0) | 50.6 (42.4- 59.9) |
| 15-29 | 27.1 (24.6-29.8 | 28.0 (25.3-30.9) | 29.5 (26.7-32.5) | 31.3 (28.5-34.4) | 28.5 (26.0-31.0) |
| 30-44 | 14.0 (12.9-15.1) | 14.2 (13.1-15.4) | 14.9 (13.7-16.1) | 14.5 (13.4-15.6) | 14.9 (13.9-16.1) |
| 45-59 | 5.8 (5.4-6.3) | 6.4 (5.9-6.9) | 6.2 (5.8-6.7) | 6.6 (6.2-7.1) | 6.7 (6.2-7.1) |
| 60-89 | 1.9 (1.8-2.1) | 2.0 (1.8-2.1) | 2.1 (1.9-2.2) | 2.00 (1.9-2.1) | 2.2 (2.1-2.3) |
| 90-104 (ref.) | 1.0 | 1.0 | 1.0 | 1.0 | 1.0 |
| ≥105 | 0.58 (0.52-0.64) | 0.52 (0.47-0.59) | 0.54 (0.48-0.61) | 0.57 (0.51-0.63) | 0.57 (0.51-0.62) |
| Missing | 0.20 (0.19-0.22) | 0.22 (0.20-0.23) | 0.25 (0.23-0.27) | 0.24 (0.23-0.26) | 0.25 (0.23-0.27) |
|  |  |  |  |  |  |
| **HbA1c among diabetics (%)** |  |  |  |  |  |
| ≥9 | 2.0 (1.8-2.3) | 1.8 (1.6-2.1) | 2.0 (1.7-2.3) | 1.9 (1.7-2.1) | 2.0 (1.8-2.2) |
| 8-8.9 | 1.5 (1.3-1.7) | 1.4 (1.2-1.6) | 1.6 (1.4-1.8) | 1.50 (1.3-1.6) | 1.41 (1.30-1.60) |
| 7-7.9 | 1.1 (0.99-1.3) | 1.05 (0.93-1.2) | 1.2 (1.0-1.3) | 1.2 (1.1-1.3) | 1.13 (1.00-1.30) |
| <6 | 1.0 | 1.0 | 1.0 | 1.0 | 1.0 |
| 6-6.9 | 0.98 (0.87-1.1) | 0.92 (0.82-1.04) | 0.96 (0.86-1.09) | 0.89 (0.80-0.98) | 0.86 (0.78-0.95) |
| Missing | 0.87 (0.77-1.0) | 0.89 (0.79-1.01) | 0.86 (0.76-0.97) | 0.86 (0.77-0.97) | 0.82 (0.74-0.91) |
|  |  |  |  |  |  |
| **Sex** |  |  |  |  |  |
| Male | 1.25 (1.2-1.3) | 1.27 (1.23-1.32) | 1.26 (1.2-1.3) | 1.36 (1.3-1.4) | 1.33 (1.3-1.4) |
| Female | 1.0 | 1.0 | 1.0 | 1.0 | 1.0 |

**Table S4. Relative deviance contribution of comorbid conditions to the risk of AKI in the population of Stockholm, 2017-2021 (%).**

|  | **2017** | **2018** | **2019** | **2020** | **2021** |
| --- | --- | --- | --- | --- | --- |
| **Age** | 6.84 | 6.82 | 6.58 | 6.45 | 7.75 |
| **eGFR level** | 4.89 | 4.13 | 3.83 | 4.12 | 4.23 |
| **Albuminuria** | 2.31 | 2.35 | 2.09 | 2.45 | 2.10 |
| **Heart failure** | 1.38 | 1.39 | 1.36 | 1.52 | 1.44 |
| **Hypertension** | 0.81 | 0.86 | 1.37 | 1.02 | 1.11 |
| **Sex** | 0.82 | 0.84 | 0.69 | 1.09 | 0.97 |
| **Previous AKI** | 0.55 | 0.84 | 0.73 | 0.66 | 0.84 |
| **Education** | 0.62 | 0.70 | 0.72 | 0.73 | 0.71 |
| **Diabetes mellitus** | 0.51 | 0.54 | 0.53 | 0.65 | 0.63 |
| **Liver disease** | 0.62 | 0.82 | 0.77 | 0.60 | 0.57 |
| **Psychiatric disorder** | 0.49 | 0.69 | 0.47 | 0.40 | 0.48 |
| **Cancer with metastasis** | 0.70 | 0.72 | 0.65 | 0.59 | 0.46 |
| **Previous fracture** | 0.43 | 0.33 | 0.34 | 0.43 | 0.43 |
| **Covid-19 infection** | - | - | - | 0.39 | 0.35 |
| **Cancer without metastasis** | 0.47 | 0.27 | 0.37 | 0.30 | 0.29 |
| **Peripheral vascular disease** | 0.35 | 0.26 | 0.33 | 0.20 | 0.20 |
| **Venous thromboembolism** | 0.09 | 0.14 | 0.11 | 0.20 | 0.18 |
| **COPD** | 0.24 | 0.14 | 0.17 | 0.09 | 0.13 |
| **Atrial fibrillation** | 0.15 | 0.19 | 0.17 | 0.11 | 0.12 |
| **Valvular disorder** | 0.16 | 0.13 | 0.20 | 0.14 | 0.12 |
| **Other chronic lung disease** | 0.07 | 0.11 | 0.10 | 0.10 | 0.11 |
| **Dementia** | 0.09 | 0.13 | 0.05 | 0.21 | 0.10 |
| **Previous colectomy** | 0.06 | 0.05 | 0.05 | 0.03 | 0.10 |
| **Pancreatitis** | 0.04 | 0.05 | 0.08 | 0.07 | 0.09 |
| **Myeloma** | 0.12 | 0.17 | 0.10 | 0.05 | 0.08 |
| **Inflammatory bowel disease** | 0.06 | 0.07 | 0.09 | 0.07 | 0.07 |
| **Rheumatic disease** | 0.12 | 0.06 | 0.08 | 0.08 | 0.06 |
| **CEVD** | 0.00 | 0.03 | 0.04 | 0.05 | 0.05 |
| **Stroke** | 0.08 | 0.09 | 0.07 | 0.06 | 0.04 |
| **Cardiac revascularization procedures** | 0.06 | 0.02 | 0.03 | 0.01 | 0.02 |
| **Kidney transplant recipient** | 0.08 | 0.00 | 0.03 | 0.01 | 0.01 |
| **Acute coronary syndrome** | 0.03 | 0.01 | 0.02 | 0.00 | 0.01 |
| **Other cardiac/aortic/valve operation** | 0.00 | 0.00 | 0.00 | 0.00 | 0.01 |
| **Other arrhythmias** | 0.01 | 0.01 | 0.00 | 0.00 | 0.00 |
| **Renal artery stenosis** | 0.01 | 0.00 | 0.00 | 0.00 | 0.00 |
| **HIV** | 0.00 | 0.00 | 0.00 | 0.01 | 0.00 |
| **Other ischemic heart disease** | 0.00 | 0.00 | 0.01 | 0.00 | 0.00 |
